# Impact of AI-assisted CXR analysis in detecting incidental lung nodules and lung cancers in non-respiratory outpatient clinics

**DOI:** 10.3389/fmed.2024.1449537

**Published:** 2024-08-07

**Authors:** Se Hyun Kwak, Kyeong Yeon Kim, Ji Soo Choi, Min Chul Kim, Chang Hwan Seol, Sung Ryeol Kim, Eun Hye Lee

**Affiliations:** ^1^Division of Pulmonology, Allergy and Critical Care Medicine, Department of Internal Medicine, Yongin Severance Hospital, Yonsei University College of Medicine, Yongin-si, Republic of Korea; ^2^Center for Digital Health, Yongin Severance Hospital, Yonsei University College of Medicine, Yongin-si, Gyeonggi-do, Republic of Korea

**Keywords:** artificial intelligence, X-rays, lung neoplasms, lung nodule, detection

## Abstract

**Purpose:**

The use of artificial intelligence (AI) for chest X-ray (CXR) analysis is becoming increasingly prevalent in medical environments. This study aimed to determine whether AI in CXR can unexpectedly detect lung nodule detection and influence patient diagnosis and management in non-respiratory outpatient clinics.

**Methods:**

In this retrospective study, patients over 18 years of age, who underwent CXR at Yongin Severance Hospital outpatient clinics between March 2021 and January 2023 and were identified to have lung nodules through AI software, were included. Commercially available AI-based lesion detection software (Lunit INSIGHT CXR) was used to detect lung nodules.

**Results:**

Out Of 56,802 radiographic procedures, 40,191 were from non-respiratory departments, with AI detecting lung nodules in 1,754 cases (4.4%). Excluding 139 patients with known lung lesions, 1,615 patients were included in the final analysis. Out of these, 30.7% (495/1,615) underwent respiratory consultation and 31.7% underwent chest CT scans (512/1,615). As a result of the CT scans, 71.5% (366 cases) were found to have true nodules. Among these, the final diagnoses included 36 lung cancers (7.0%, 36/512), 141 lung nodules requiring follow-up (27.5%, 141/512), 114 active pulmonary infections (22.3%, 114/512), and 75 old inflammatory sequelae (14.6%, 75/512). The mean AI nodule score for lung cancer was significantly higher than that for other nodules (56.72 vs. 33.44, *p* < 0.001). Additionally, active pulmonary infection had a higher consolidation score, and old inflammatory sequelae had the highest fibrosis score, demonstrating differences in the AI analysis among the final diagnosis groups.

**Conclusion:**

This study indicates that AI-detected incidental nodule abnormalities on CXR in non-respiratory outpatient clinics result in a substantial number of clinically significant diagnoses, emphasizing AI’s role in detecting lung nodules and need for further evaluation and specialist consultation for proper diagnosis and management.

## 1 Introduction

Recent advancements in artificial intelligence (AI) have led to the widespread integration of various AI technologies in healthcare settings. Among these, AI applications for interpreting chest X-rays (CXRs) have been increasingly utilized (1–4). CXRs are fundamental diagnostic tools that are routinely used in primary care and referral hospitals because of their accessibility and ease of use. They are commonly used for patients with respiratory symptoms and as part of routine outpatient and inpatient examinations, preoperative assessments, and emergency room visits. Several previous studies have demonstrated the benefits of AI in detecting malignant lung nodules on CXRs (5, 6). Despite the widespread use of CXRs in clinical settings, obtaining immediate radiological interpretations remains challenging, particularly in outpatient and emergency environments. This limitation has driven the increased use of AI as an instant screening tool, helping bridge the gap between image acquisition and diagnosis (7–9).

Among the AI-detected CXR abnormalities, those accompanied by respiratory symptoms, such as pneumothorax and acute respiratory infections, are easier to detect based on the patient’s symptoms. In contrast, incidental lung nodules, which often present without respiratory symptoms, are particularly challenging for identification and accurate diagnosis. These nodules can lead to missed lung cancer and delayed diagnosis (10–12), eventually affecting patient outcomes. Therefore, the discovery of an appropriate diagnostic approach for incidental lung nodules is crucial for patient prognosis. Although the role of AI in enhancing diagnostic accuracy is well-documented (13–15), there is a lack of research on the actual diagnostic approaches and interventions implemented for patients with abnormalities identified by AI in real-world clinical settings. Therefore, it is essential to understand how AI-flagged abnormalities in CXRs influence diagnostic processes and treatment interventions in real-world healthcare settings.

This study aimed to investigate the diagnostic processes and clinical outcomes of patients with AI-detected lung nodule abnormalities on CXR in non-respiratory outpatient clinics and to explore the clinical significance of these AI abnormalities in patient care.

## 2 Material and methods

### 2.1 Patients and clinical data

**FIGURE 1:**
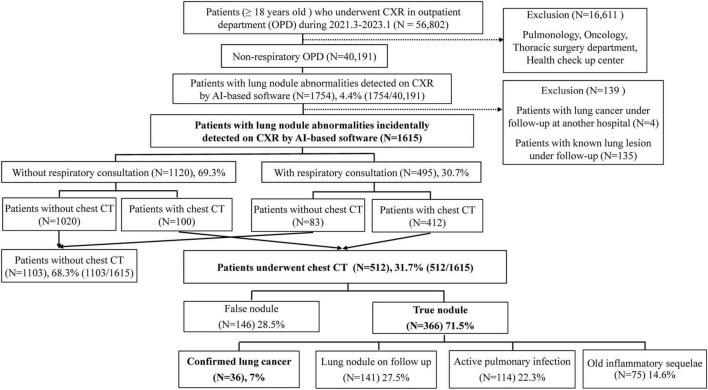
Flowchart of study patient enrollment. AI, artificial intelligence; CXR, chest radiograph; CT, computed tomography.

Patients (≥ 18 years old) who underwent CXR at the outpatient clinic between March 2021 and January 2023 at Yongin Severance Hospital were retrospectively reviewed (Figure 1). The hospital implemented AI software for all CXR performed, providing immediate reports of abnormal findings at the time the CXRs were performed. Among them, we included patients with lung nodules detected on CXR by AI in the outpatient clinic after excluding those related to the lung abnormalities department (pulmonology, thoracic surgery, and oncology), and health check-up center. In cases where a patient underwent multiple CXR, we used the first CXR as the subject of analysis. The medical records of the patients’ diagnostic workup, whether chest computed tomography (CT) was performed, final diagnosis, and diagnosis of lung cancer were reviewed. Lung cancer stage was assessed according to the 8th edition of the TNM classification (16). The research protocol was approved by the Institutional Review Board (IRB) of Yongin Severance Hospital (IRB No. 9-2024-0087). The requirement for informed consent was waived because of the retrospective nature of the study.

### 2.2 AI-based CXR analysis

Commercially available AI-based lesion detection software (Lunit INSIGHT for Chest Radiography, version 3.1.2; Lunit Inc., Republic of Korea) has been used for all CXRs in our hospital since March 2021. The software can detect eight different lesion types (nodule, consolidation, pneumothorax, pneumoperitoneum, fibrosis, atelectasis, cardiomegaly, and pleural effusion). Lesion location was displayed in the chest radiographs by a grayscale contour map when the abnormality score (probability of an abnormal lesion existing) was above the preset operating point of 15% (17, 18). The radiograph classification and nodule detection performances are reported to have an area under the curve (AUC) in the range of 0.92–0.99 (19). The earlier version detects the nodule only, whereas INSIGHT CXR 3 is equipped with an extended list of detectable abnormalities including lung nodule, consolidation, and pneumothorax with an accuracy of 97–99% (20). The analyzed AI results were automatically attached to the original CXR image as a secondary file, allowing the attending physician to immediately check the results. We extracted each abnormality score and lesion type from the AI server by uploading digital imaging and communications in medicine (DICOM) images of the CXR to the server.

### 2.3 Classification of true lung nodules

For patients with AI nodule score of 15% or higher on CXR, the actual number of nodules was analyzed only in those who underwent chest CT within 3 months after CXR was performed. An actual nodule in the lesion detected as a nodule by AI on chest CT was defined as a true nodule; if there was no actual nodule on chest CT, it was defined as a false nodule. The final diagnoses of the nodules were classified into the following four categories: (1) patients ultimately diagnosed with lung cancer; (2) nodules that exist but are not confirmed as lung cancer and require follow-up; (3) active pulmonary infections such as pneumonia, pulmonary tuberculosis (TB), and nontuberculous mycobacteria (NTM); and (4) old inflammatory sequelae. Old inflammatory sequelae are characterized by the presence of calcified nodules, pleural thickening, fibrosis, volume loss, and parenchymal bands. The presence of nodules on chest CT was determined based on the official interpretation by radiologists, and the final diagnosis was verified through a review of the images by two pulmonologists, SHK and EHL, with 5 and 8 years of experience, respectively. Medical records for diagnostic work-up and final diagnosis were reviewed until April 2023 and 3 months after the last CXR was performed.

### 2.4 Statistical analysis

Statistical analyses were performed using R program (version 4.4.0, Foundation for Statistical Computing, Vienna, Austria; packages: survival, rms, compareC, and pec). Values are presented as mean with standard deviation (SD) or median with interquartile range (IQR). The ggplot2 library facilitated the generation of plots to compare the AI abnormality scores between true and false nodules across the final diagnosis groups. The mean difference was compared using *t*-test, and a *p*-value < 0.05 was considered significant for all analyses.

## 3 Results

### 3.1 Study patients

**FIGURE 2:**
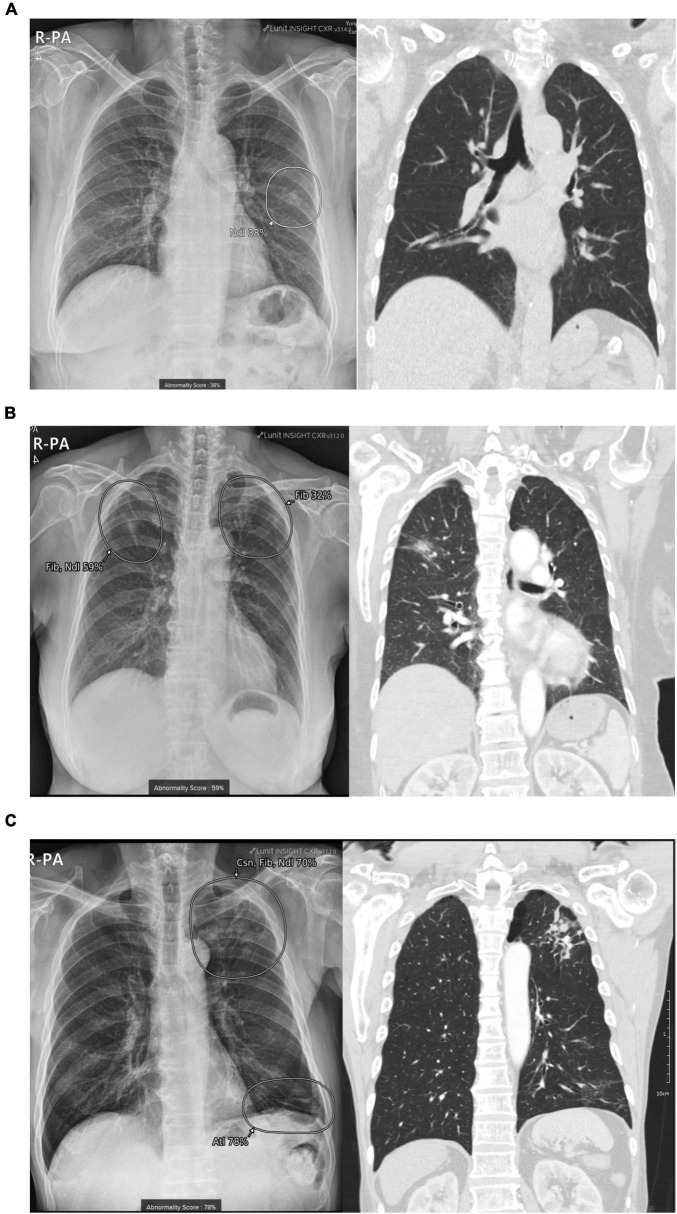
Examples of patient cases. **(A)** False-positive nodule detected on CXR by AI: A 75-year-old man underwent a CXR examination at the cardiology outpatient clinic, which suggested a nodule in the left middle lung field (AI nodule score: 38%). Subsequent chest CT revealed no significant abnormalities, and the nodule was identified as a rib or a vascular shadow. **(B)** True-positive nodule detected on CXR by AI, lung cancer: A 71-year-old woman underwent a preoperative examination for spinal surgery, which suggested fibrosis combined with a nodule in the right upper lung field. A subsequent chest CT scan revealed an approximately 3 cm part-solid nodule. The final pathological diagnosis after surgery confirmed stage IA adenocarcinoma (Nodule score: 59%; Fibrosis score: 26%). **(C)** True-positive nodule detected on CXR by AI and acute pulmonary infection: A 75-year-old female patient underwent CXR at the gastroenterology outpatient clinic, where AI suggested combined findings of consolidation, fibrosis, and a nodule in the left upper lung field. Subsequent chest CT revealed multiple centrilobular nodules, and a sputum test was performed to diagnose active pulmonary TB (AI Nodule score, 54%; consolidation score, 24%; fibrosis score, 70%). AI, artificial intelligence; CXR, chest radiograph; Ndl, nodule; Csn, consolidation; Fib, fibrosis.

Between March 2021 and January 2023, a total of 56,802 patients (≥ 18 years old) underwent CXR in the outpatient department (OPD) (Figure 1). After excluding pulmonology, oncology, thoracic surgery department, health-up center, 40,191 patients with CXR were analyzed. Among these patients, 1,754 (4.4%) had lung nodule abnormalities detected on CXR by AI software (Figure 1). A total of 1,615 patients were included in the final analysis after excluding 135 patients with known lung lesions and 4 patients who were being followed up for lung cancer at another hospital. Out of these, 30.7% (495/1,615) underwent respiratory consultation and 69.3% (1,120/1,615) did not undergo respiratory consultation. Among the 495 patients who underwent respiratory consultation, 412 performed additional chest CT, and 100 out of 1,120 patients who did not undergo respiratory consultation also performed chest CT within 3 months of CXR, making a total of 512 patients (31.7%, 512/1,615). Out of 512 patients with chest CT, 146 patients (28.5%) were considered false positives because the nodule was not actually present on chest CT. The remaining 366 patients (71.5%) had actual nodules discovered on chest CT, and 36 patients, which is 7% of all patients who underwent chest CT, were ultimately diagnosed with lung cancer. Additionally, 141 patients (27.5%) were classified as having lung nodules requiring follow-up; 114 patients (22.3%) had pulmonary infections such as pneumonia, pulmonary TB, and NTM; and the remaining 75 patients (14.6%) had old inflammatory sequelae (Figure 1). Table 1 presents the characteristics of each diagnostic group. Compared to the group ultimately diagnosed with lung cancer (A), the old inflammatory sequelae group (D) had a significantly higher history of old TBc. Additionally, the nodule size and AI nodule score in group A were significantly higher than those in the other groups (*p* < 0.001, all). Figure 2 shows examples of patients who underwent chest CT after suspected lung nodules were detected on CXR by AI.

**TABLE 1:** The characteristics of the 512 patients who underwent chest CT.

	A	B	C	D	E	*P*-value			
**Characteristic**	**Lung cancer (*n* = 36)**	**Other lung nodules (*n* = 141)**	**Pulmonary infection (*n* = 114)**	**Old inflammatory sequelae (*n* = 75)**	**False nodule (*n* = 146)**	**A vs. B**	**A vs. C**	**A vs. D**	**A vs. E**
Age, year, IQR	74.5 (69.2–83.2)	75 (66–81)	73 (63.2–80.8)	68 (60.5–76)	71.5 (60–80)	0.572	0.41	0.019	0.109
Sex, male, *n* (%)	24 (66.7)	81 (57.4)	69 (60.5)	33 (44)	70 (49.7)	0.415	0.642	0.042	0.068
Ever smoker, *n* (%)	15 (41.7)	52 (36.9)	32 (28.1)	21 (28.0)	40 (27.4)	0.737	0.184	0.221	0.142
**Comorbidity, *n* (%)**									
HTN	24 (66.7)	79 (56.0)	59 (51.8)	45 (60.0)	82 (56.2)	0.334	0.169	0.639	0.339
DM	14 (38.9)	42 (29.8)	43 (37.7)	21 (28.0)	39 (26.7)	0.397	1.0	0.348	0.217
Old Tbc	5 (13.9)	17 (12.1)	13 (11.4)	36 (48.0)	13 (8.9)	0.989	0.916	0.001	0.558
Nodule characteristics*									
GGO/subsolid	7 (19.4)	36 (25.5)	51 (44.7)	0	–	0.588	0.001	< 0.001	–
Solid	29 (80.6)	105 (74.5)	53 (46.5)	0	–				
Calcification/fibrotic scar	0		10 (8.77)	75 (100)	–				
Nodule size*									
≤ 1 cm	0	106 (75.2)	111 (97.4)	71 (94.7)	–	< 0.001	< 0.001	< 0.001	–
> 1 cm, ≤ 2 cm	7 (19.4)	20 (14.2)	0	1 (1.3)	–				
> 2 cm, ≤ 3 cm	9 (25)	8 (5.7)	1 (0.9)	2 (2.7)	–				
> 3 cm	20 (55.6)	7 (4.9)	2 (1.8)	1 (1.3)	–				
**AI abnormality score, mean ± SD**									
Nodule	56.7 ± 29.7	34.5 ± 21.9	28.1 ± 15.3	36 ± 21.9	29.4 ± 20.6	< 0.001	< 0.001	< 0.001	< 0.001
Consolidation	30.3 ± 35.8	23.9 ± 30.7	57.2 ± 33.8	19.8 ± 26.0	38.0 ± 36.1	0.331	< 0.001	0.121	0.255
Fibrosis	21.9 ± 23.9	23.1 ± 28.7	31.4 ± 32.0	41.0 ± 34.8	23.3 ± 31.1	0.792	0.059	0.001	0.767
Atelectasis	7.21 ± 10.6	9.55 ± 16.7	14.0 ± 19.4	10.1 ± 17.1	14.5 ± 21.9	0.302	0.009	0.285	0.005
Pleural effusion	21.8 ± 33.6	10.8 ± 23.5	22.7 ± 34.7	13.7 ± 26.1	18.0 ± 32.6	0.069	0.895	0.206	0.539

*For multiple nodules, characteristics of the nodule with the maximal size. Values are presented as medians with interquartile ranges (IQR) or numbers with percentages. IQR, interquartile range; SD, standard deviation; HTN, hypertension; DM, diabetes; Tbc, tuberculosis; GGO, ground glass opacity.

### 3.2 Difference in AI lung nodule score between groups during diagnostic work-up

**FIGURE 3:**
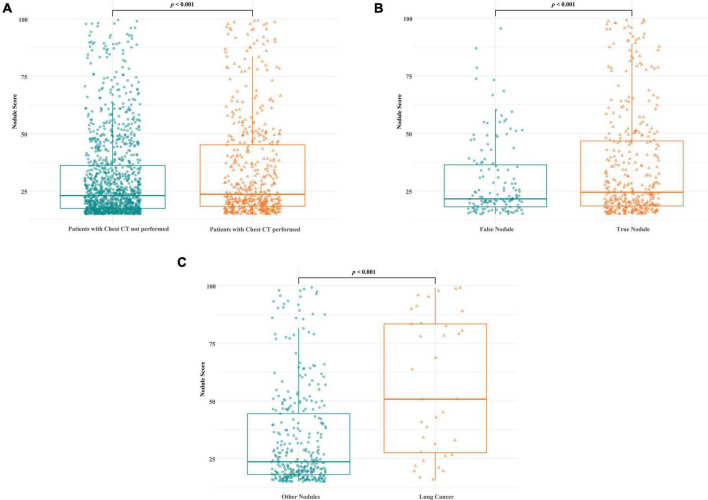
Comparison of AI nodule scores on CXR among each group during the diagnostic workup process. **(A)** The mean AI lung nodule score was significantly higher in the group of patients who underwent chest CT than in those who did not [**(A)**, mean 30.3 vs. 33.9, *p* < 0.001]. **(B)** Among the patients who underwent CT, the mean AI nodule score was significantly higher in the true nodule group than in the false nodule group (mean 29.4 vs. 35.7, *p* < 0.001). **(C)** Among the patients with true nodules, the mean AI nodule score was significantly higher in those diagnosed with lung cancer than in those diagnosed with other conditions (mean 33.4 vs. 56.7, *p* < 0.001).

Figure 3 shows the differences in the lung nodule AI scores between each group during the lung nodule diagnostic workup process. Among all patients with abnormal lung nodule scores on CXR, the mean AI lung nodule score was significantly higher in the group of patients who underwent chest CT than in those who did not (Figure 3A, mean 30.3 vs. 33.9, *p* < 0.001). In addition, among the patients who underwent chest CT, the mean AI nodule score of the true nodule group was significantly higher than that of the false nodule group (Figure 3B, mean 29.4 vs. 35.7, *p* < 0.001). Additionally, the AI nodule scores of the 36 patients ultimately diagnosed with lung cancer showed significantly higher mean values than those of patients diagnosed with other nodules (Figure 3C, mean 33.4 vs. 56.7, *p* < 0.001).

### 3.3 Difference in AI score between different final diagnosis groups

**FIGURE 4:**
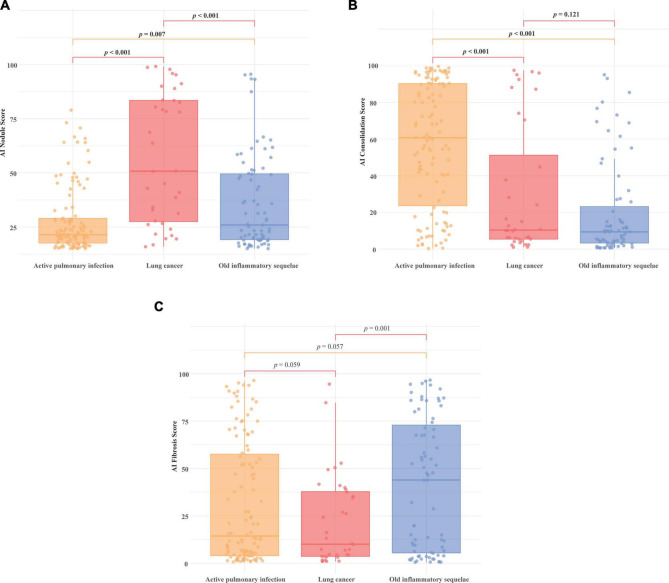
Comparison of AI nodule, consolidation, and fibrosis scores by final diagnosis group. Comparison of AI scores on CXR among the three groups diagnosed with active pulmonary infection (*n* = 114), lung cancer (*n* = 36), and old inflammatory sequelae (*n* = 75). The AI scores for **(A)** nodule, **(B)** consolidation, and **(C)** fibrosis are shown for each group. **(A)** The mean nodule score was significantly higher in lung cancer patients than in the active pulmonary infection (*p* < 0.001) and old inflammatory sequelae groups (*p* < 0.001) (mean 28.1 vs. 56.7 vs. 36.0, respectively). **(B)** The mean consolidation score of active pulmonary infection was statistically higher than lung cancer (*p* < 0.001) and old inflammation sequelae (*p* < 0.001), respectively (mean 57.2 vs. 30.3 vs. 19.8). **(C)** Fibrosis score: The old inflammatory sequelae group showed a statistically higher mean fibrosis score than lung cancer (*p* = 0.001), and there was no statistical difference between the active pulmonary infection and old inflammatory sequelae group (*p* = 0.057) (mean 31.4 vs. 21.9 vs. 41.0).

Figure 4 shows the results of the comparison of AI scores on CXR among the three groups diagnosed with active pulmonary infection (*n* = 114), lung cancer (*n* = 36), and old inflammatory sequelae (*n* = 75). Depending on the patient’s final diagnosis, nodules, consolidation, and fibrosis may coexist; however, the nodule abnormality score was statistically higher in lung cancer patients than in the active pulmonary infection (*p* < 0.001) and old inflammatory sequelae patients (*p* < 0.001) (Figure 4A, mean 28.1 vs. 56.7 vs. 36.0). In the case of the mean consolidation score, active pulmonary infection was statistically higher than lung cancer (*p* < 0.001) and old inflammatory sequelae (*p* < 0.001) (Figure 4B, mean 57.2 vs. 30.3 vs. 19.8). Finally, in the case of AI fibrosis scores, the old inflammatory sequelae group showed a statistically higher mean level than lung cancer (*p* = 0.001); however, there was no statistically significant difference from active pulmonary infection (0.057) (Figure 4C, mean 31.4 vs. 21.9 vs. 41.0).

### 3.4 Characteristics of patients diagnosed with lung cancer

Table 2 shows the baseline characteristics of the 36 patients ultimately diagnosed with lung cancer. The median age was 74.5 years, and 66.7% of patients were male. Approximately 55.5% of patients had confirmed adenocarcinoma, and 3 patients were diagnosed with lung cancer via a multidisciplinary approach without pathologic confirmation. Overall, 33.3% of the total patients were finally confirmed as stage I, and surgical treatment was performed in 36.1% of the total patients. Regarding the AI abnormality score, the nodule score showed the highest mean value; however, the mean values of consolidation, fibrosis, and pleural effusion also showed abnormal values > 15%, indicating that they can be accompanied by abnormal findings other than nodules (Table 1).

**TABLE 2:** Characteristics of 36 patients diagnosed with lung cancer.

	Total (*n* = 36)
Age, year, IQR	74.5 (39.25–83.25)
Sex, male, *n* (%)	24 (66.7)
**Subtype, *n* (%)**	
Adenocarcinoma	20 (55.5)
Squamous cell carcinoma	8 (22.2)
SCLC	5 (13.8)
Not confirmed (radiologic diagnosis)	3 (8.3)
**Pathologic stage, *n* (%)**	
I	12 (33.3)
II	3 (8.3)
III	5 (13.8)
IV	16 (44.4)
**Treatment**	
Surgery	13 (36.1)
SBRT	5 (13.8)
CCRT	2 (5.5)
Chemotherapy	13 (36.1)
Palliative care only	3 (8.3)

Values are presented as medians with interquartile ranges (IQR) or numbers with percentages. IQR, interquartile range; SCLC, small cell lung cancer; SBRT, stereotactic body radiotherapy; CCRT, concurrent chemoradiotherapy.

## 4 Discussion

In this study, we investigated the clinical processes of incidental lung nodules detected on CXR in a non-respiratory outpatient setting. The results showed that despite the detection of lung nodule abnormalities by AI, only 30.7% of patients underwent respiratory consultation and chest CT was performed in 31.7% of the cases. Among the patients who underwent further chest CT workup, 71.5% were found to have true nodules and 7% were diagnosed with incidental lung cancer. Additionally, among the 512 patients who underwent chest CT after showing abnormal nodule scores on CXR by AI, 291 patients (56.8%) required additional follow-up or therapeutic intervention (36 lung cancer, 141 lung nodules requiring follow-up, 114 active pulmonary infections, Figure 1).

Lung cancer remains the leading cause of cancer-related mortality worldwide (21), with the highest incidence among men and fifth among women in Korea (22, 23). Despite advancements in diagnostic technology, the role of chest radiography in the early detection of lung cancer remains unclear. Currently, low-dose chest CT scans are recommended for early detection; however, their application is mostly confined to high-risk groups (24, 25). This limitation is particularly concerning for lung cancer screening in women, who account for one-third of all lung cancer cases and for the increasing number of lung cancers in nonsmokers. Recent large-scale studies conducted in Korea have highlighted the potential benefits of chest radiography in identifying early stage lung cancer (26). These studies have shown a significant rate of early stage lung cancer detection in patients who underwent chest radiography prior to their diagnosis. Notably, women who participated in chest X-ray screenings exhibited about a 10% reduction in mortality rate, suggesting a critical need for further research into the effectiveness of chest X-rays in detecting pulmonary nodules and lung cancer (26). Considering that the incidence of lung cancer in large-scale RCTs targeting high-risk groups is less than 1% (24, 25), the finding that 7% of patients who underwent chest CT based on AI-detected lung nodule abnormalities on CXR performed in non-respiratory departments were diagnosed with lung cancer was notably high. Furthermore, among the 512 patients who underwent chest CT based on AI findings, not only was lung cancer detected, but 56.8% showed clinically significant results, including lung nodules requiring follow-up and pulmonary infections such as TB, pneumonia, and NTM. This underscores the importance of a further workup for CXR nodule abnormalities. This demonstrates the clinical effectiveness of the AI CXR nodule software.

While AI abnormal findings on CXR cannot by themselves provide a definitive diagnosis, they can serve as a basis for further workup, such as chest CT scans or respiratory consultations. This facilitates the detection of respiratory diseases and lung cancer that might otherwise be missed in asymptomatic patients, especially in non-respiratory department. However, in our study, 28.5% of patients who underwent chest CT were found to have false positive nodules, raising concerns about the potential risk of overexposure to unnecessary tests. The AI’s ability to assign abnormality scores is crucial in early detection and decision-making, potentially improving patient outcomes. However, the potential for false positives and subsequent excessive testing must be carefully managed. Additional research to evaluation of the cost-effectiveness analysis of the actual implementation of AI CXR and explore ways to reduce false positives is necessary.

On the other hand, 68.3% of the patients with AI-detected lung nodule score abnormalities on CXR did not undergo additional workup. Although AI is predominantly used in medical environments, physicians who are not specialists in the relevant field may not fully understand the abnormalities detected by AI. In the case of CXR, the understanding of AI-detected abnormalities may be limited among physicians who are not radiologists or specialists in respiratory or thoracic medicine. Therefore, even if AI abnormalities are detected, there may be instances in which non-radiologists or non-respiratory specialists may not recognize these abnormalities owing to a lack of understanding of the software. In our institution, there are no alert alarms or critical value reports (CVR) for CXR abnormalities directed at attending physicians. Therefore, it is difficult to determine whether non-respiratory physicians are aware of AI-detected abnormalities. Although there have been numerous studies on the diagnostic accuracy of AI-detected abnormalities and the efficiency of AI in reducing radiologists’ reading time and effort (27–29), studies on how AI-detected abnormal nodule findings in clinical settings influence physicians’ diagnostic processes are rare. Our study differs from previous research by examining how diagnostic workups are conducted in clinical settings when AI detects abnormal nodule findings. In our study, a large proportion of patients with AI-detected abnormal nodules did not receive further workup, highlighting the necessity of establishing proper diagnostic processes. This includes providing AI education to attending physicians, implementing the alert alarm system for AI-detected abnormalities, and ensuring appropriate referrals to specialists.

This study had several limitations. Firstly, as a retrospective study conducted at a single institution, it is challenging to generalize the findings. Additionally, for a large proportion of patients who did not receive further workup, it is difficult to determine the exact reasons, and we lack information on the patients’ demographic characteristics that might have influenced the decision for additional workup. Additionally, the study did not include a normal control group, making it difficult to assess the AI’s performance and accuracy. Despite its limitations, this study is valuable as it investigates how commercially approved AI software for CXR nodule abnormalities is applied in clinical practice and provides insights into the diagnostic process and outcomes.

In conclusion, this study indicated that AI-detected incidental nodule abnormalities on CXR in non-respiratory outpatient clinics lead to a substantial number of clinically significant diagnoses, including lung cancer and respiratory infections, highlighting the potential role of AI in identifying abnormal lung nodules. When AI detects nodule abnormalities on CXR, clinical attention and further evaluation, such as specialist consultation and additional diagnostic workup, are necessary to ensure proper diagnosis and management. Moreover, to effectively connect AI-detected abnormalities to patient diagnostic strategies, the integration of alert signals into AI systems may be necessary.

## Data availability statement

The original contributions presented in this study are included in the article/supplementary material, further inquiries can be directed to the corresponding author.

## Ethics statement

The studies involving humans were approved by the Institutional Review Board of Yongin Severance Hospital. The studies were conducted in accordance with the local legislation and institutional requirements. The ethics committee/institutional review board waived the requirement of written informed consent for participation from the participants or the participants’ legal guardians/next of kin because of the retrospective nature of the study and the use of anonymized clinical data. Written informed consent was not obtained from the individual(s) for the publication of any potentially identifiable images or data included in this article because the requirement for informed consent was waived by the IRB of Yongin Severance Hospital because of the retrospective nature of the study and the use of anonymized clinical data.

## Author contributions

SHK: Formal analysis, Writing – original draft. KK: Data curation, Formal analysis, Writing – review & editing. JC: Writing – review & editing. MK: Writing – review & editing. CS: Writing – review & editing. SRK: Writing – review & editing. EL: Conceptualization, Data curation, Formal analysis, Funding acquisition, Writing – original draft, Writing – review & editing.
